# Associated factors and principal pathophysiological mechanisms of type 2 diabetes mellitus

**DOI:** 10.3389/fendo.2025.1499565

**Published:** 2025-05-09

**Authors:** Luyao Wang, Lianxin Li, Jiaxin Liu, Changting Sheng, Maoyi Yang, Zhipeng Hu, Rensong Yue

**Affiliations:** Department of Endocrinology, Hospital of Chengdu, University of Traditional Chinese Medicine, Chengdu, China

**Keywords:** type 2 diabetes mellitus, pathogenic mechanism, insulin resistance, mitochondrial function, obesity, gut microbiota

## Abstract

Type 2 diabetes mellitus(T2DM) as a common chronic disease with an increasing prevalence worldwide that poses a great threat to individual health, and is characterized by chronic hyperglycemia resulting from insulin resistance (IR) coupled with β-cell dysfunction. Mitochondrial dysfunction, obesity, gut microbiota, oxidative stress and inflammation have emerged as a significant contributor to the etiology of T2DM, affecting various metabolic processes critical for glucose homeostasis. This short review underscores their role in enhancing T2DM-related molecular mechanisms and explores recent advancements in diabetic management, further highlights the importance of personalized care plans to address the complexities of the T2DM and aims to improve patient quality of life and long-term health outcome.

## 1 Introduction

Type 2 diabetes mellitus (T2DM) is a renowned overgrowing endocrine metabolic disease, and occurs as a result of insulin resistance (IR) and inadequate insulin production, resulting in hyperglycemia, presenting a substantial burden on global healthcare systems (1, 2). IR is characterized by an impaired cellular response to insulin stimulation in peripheral tissues, such as the liver, skeletal muscle and adipose tissue.

As T2DM advances, progressive beta cell dysfunction occurs, results in relative insulin deficiency of insulin secretion (3). Besides that, mitochondrial dysfunction, obesity, gut microbiota, oxidative stress and inflammation are also implicated in the etiology of T2DM,these different pathogenic factors underscore the urgent need for multifactorial approach that address not only control of hyperglycemia but also the broader implications and side-effects impact of overall health and well-being (4). T2DM management, pharmacological interventions alone is insufficient, lifestyle modifications, weight management, regular physical activity and patient self-management are critical to advance diabetic care (5). In fact, by addressing multifactorial characteristics of onset and development in T2DM, a focus on individualized care and prevent the risk of complications must consider patient-specific needs, effective and sustainable treatment preferences, comorbidities, ultimately optimizing patient overall quality of life and long-term health outcomes (6). Lastly, we discuss the latest therapeutic innovations, like gene therapy, regenerative medicine and identification of omics-related biomarkers, aid in gaining a deeper comprehension of holistic care and precision medicine among diabetic patients.

## 2 Mechanisms and pathogenic factors of IR

### 2.1 Mitochondrial dysfunction

Mitochondria, commonly known as the “powerhouses” of cells, containing their own double-stranded DNA (mtDNA), which encodes 13 polypeptides constitute mostly part of the electron transfer chain (ETC) (7). In fact, the mitochondrial ETC is the site of oxidative phosphorylation (OXPHOS), which is key in glucose metabolism and the biggest net producer of ATP in mammalian cells (8). Mitochondria play a crucial role in apoptosis, signaling, oxidation processes, and cellular energy consumption and balance, as well as are vital for the proper functioning and survival of pancreatic β-cells (9). When mitochondria are dysfunctional, uncontrolled fission or fusion occurs, like impaired mitochondrial fusion or excessive mitochondrial fission, resulting in impaired mitochondrial dynamics and mitochondrial fragmentation. Reactive oxygen species (ROS) are next to the location of the mtDNA and produced by OXPHOS, this makes the mtDNA highly susceptible to oxidative damage, thus increasing the probability of mutations and further disturbing mitochondrial energy metabolism (10, 11). Meanwhile, this oxidative damage compromises mETC function and worsens energy failure, in addition to oxidative stress and dysfunctional mitochondria, which have been implicated in the part pathogenesis of various diseases, including DM (12). Peroxisome proliferator-activated receptors (PPARs) agonists, like pioglitazone is a promising thiazolidinediones and restores mitochondrial function in patients with T2DM (13). Glimins represent a new class of oral glucose-lowering drugs, primarily targeting the mitochondrial respiratory chain complex to reduce the production of ROS and prevent mitochondrial meability transition pore opening, thereby restoring mitochondrial function in skeletal muscle, liver, and pancreas of diabetic patients (14, 15).

### 2.2 Mitochondrial biogenesis

**Figure 1:**
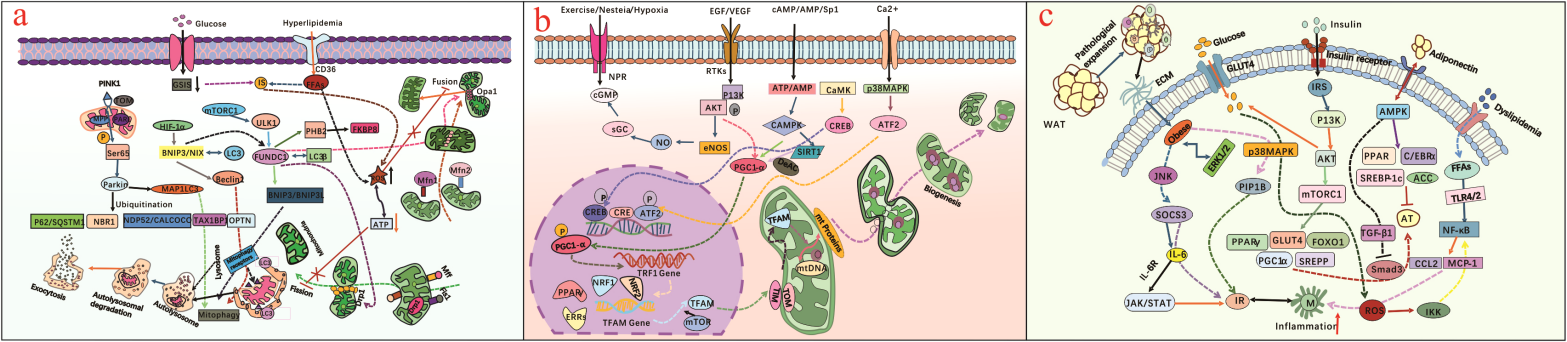
**(a)** illustrates mitochondrial dysfunction (abnormal mitochondrial fusion or excessive mitochondrial fission) triggers mitochondrial fragmentation, anomalous mitophagy, overproduced ROS, reduced ATP synthesis, dysregulated mitochondrial dynamics, further impairs cellular metabolism and contributes to T2DM progression; **(b)** presents the extracellular environment cues affect mitochondrial biogenesis and dynamics, as dysfunctional mitochondria fail to adapt to changing metabolic demands, leading to cellular dysfunction and T2DM; **(c)** presents adipocyte-induced obesity is significant in T2DM pathogenesis, and obesity-related dyslipidemia, inflammation and insistent hyperglycemia condition regulate IR through different molecular pathway.

Mitochondrial biogenesis is implicated in cells grow and mitochondrial responses to environmental cues and metabolic demands, which is a key feature of mitochondrial function, its dysregulation contributes to the development and progression of T2DM (16). Transcriptional coactivators peroxisome proliferator-activated receptor gamma coactivator-1 alpha (PGC-1α), initiates mitochondrial biogenesis pathway by sequential activations of nuclear transcription factors, including nuclear respiratory factors (NRF1/2), estrogen-related receptor-alpha(ERR-α), followed by mitochondrial transcription factor A (TFAM) and mtDNA replication and transcription. Dysregulation of PGC-1α activity leads to impaired mitochondrial biogenesis and gene expression in oxidative phosphorylation and metabolic disorders (17). Sirtuin 1 (Sirt1) is a nicotinamide adenine dinucleotide (NAD+) -dependent histone deacetylase that plays a significant role in modulating PGC-1α activity, promoting mitochondrial biogenesis and renewal (18). Inflammatory cytokines, calcium (Ca^2+^) regulation and metabolic stressors, such as hyperglycaemia and dyslipidaemia, are also linked to inhibit mitochondrial biogenesis and promote onset of IR (19). Substances such as 5-Aminoimidazole-4-carboxamide ribotide, GW501516, and various natural compounds present in epicatechin, have been identified as potential pharmacological via stress kinases, transcription factors and peroxisome proliferator-activated receptors (PPARs) activation, as well as mitochondrial function restoration to alleviate metabolic abnormalities (20). Besides, mitochondrial gene therapy has emerged as an innovative tool hold significant promise for restoring mitochondrial function and enhancing cellular bioenergetics (21). Addressing mitochondrial dysfunction and T2DM crosstalk is crucial for understanding the biochemistry mechanisms involved in overproduction of ROS, reduced ATP synthesis, dysregulated mitochondrial dynamics, poor mitochondrial biogenesis, and impaired mitochondrial gene expression, resulting in imbalance between energy generation and consumption, further exacerbate metabolic disorders, which may be exactly identified the relationship between mitochondrial dysfunction and molecular mechanisms underlying T2DM is multifaceted and complex (16)(**Figures 1a, b**).

### 2.3 Obesity

An long-term imbalance in energy between intake and expenditure is a key characteristic of individuals with obesity, and then chronic exposure to hyperglycemia, dyslipidemia, glucotoxicity impairs β-cell function and viability, leading to progressive deterioration of insulin secretion capacity (22, 23). Brown adipose tissue (BAT) is a distinct type of adipose tissue that dissipates energy and plays a natural anti-obesity role. White adipose tissue (WAT) primarily stores energy. Disturbances in BAT and WAT homeostasis have been associated with certain microbial imbalances and onset of obesity (24). Given that obesity has been closely implicated in the pathogenesis of T2DM (**Figure 1c**). Browning agents, such as cold exposure, β-3 adrenergic receptor agonists (CL 316243, BRL 26830A), short-chain fatty acids (butyrate, propionate, acetate), are compounds that can promote the conversion of WAT into BAT, may hold promise as a target for treating and preventing T2DM (25, 26). Besides, as reciprocal causation between obesity and T2DM, significant progress in the pharmacological treatment revolve around insulin resistance-mediated obesity. Metformin is the preferred first line antidiabetic drug, exerts its therapeutic effects by increasing insulin sensitivity, glucose uptake by activating adenosine monophosphate activated protein kinase, promoting weight loss, improving lipid profiles, as well as modulating mitochondrial dynamics and biogenesis, thus contributing to its metabolic benefits in T2DM onset (27). Sodium-glucose cotransporter 2 (SGLT2) inhibitors, such as dapagliffozin and empagliffozin, have emerged as promising therapeutic agents for antihyperglycemic management (28). These substances reduce body weight, initially through a direct effect, and subsequently by shifting substrate utilization from carbohydrates to lipids, thereby reducing body fat, including visceral and subcutaneous fat (29, 30). Gastric inhibitory polypeptide (GIP) and glucagon-like peptide 1 (GLP-1) are secreted by cells in the human gut after food intake and regulate insulin release by pancreatic β-cells, and involved in blood sugar homeostasis. Tirzepatide acts on the GIPR/GLP-1R receptors, and is a recently developed drug useful in the treatment of T2DM and for weight loss, studies show its role in improving circulating levels of adiponectin, eventually influences lipid and glucose metabolism (31, 32). What needs to be emphasized that each drug has specific mechanisms and potential side effects, necessitating the significance of personalized treatment plan. Besides that, bariatric surgery like sleeve gastrectomy, one-anastomosis gastric bypass, and Roux-en-Y gastric bypass have the potential to induce remission of T2DM-related obesity (33).

### 2.4 Gut microbiota

**Figure 2:**
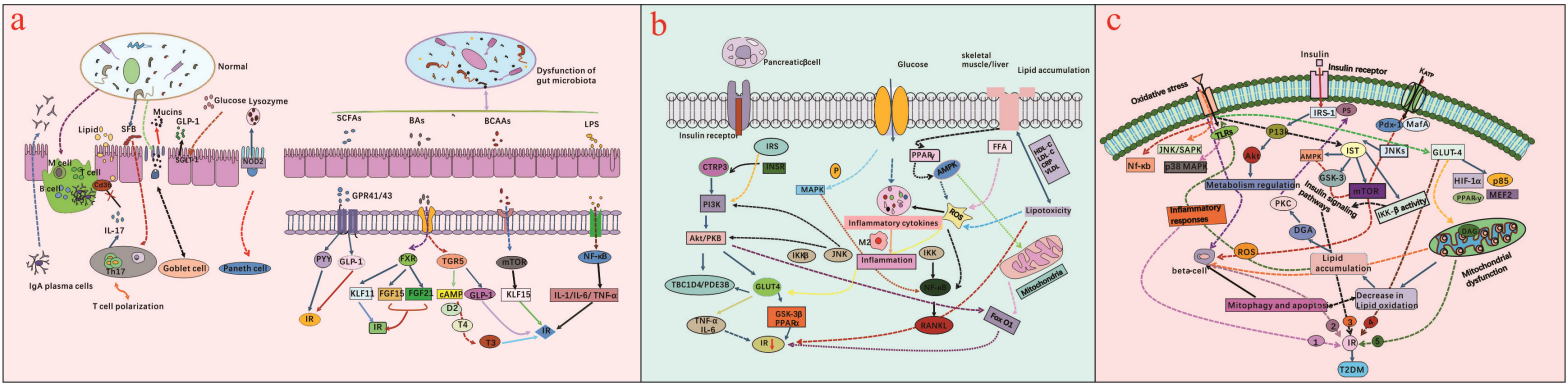
**(a)** illustrates dysbiosis of gut microbiota is related to LPS-induced inflammatory pathways, as well as BCAAs, SCFAs and BAs disrupt metabolic pathways, eventually triggers IR by impairing the gut mucosal barrier and increasing intestinal permeability; **(b)** presents inflammatory pathways impacting IR and possible action, and illustrates chronic hyperglycemia, oxidative stress, lipid metabolism and B-cell dysfunction can activate pro-inflammatory pathways, further exacerbate IR in T2DM; **(c)** presents oxidative stress-induced insulin resistance-related signaling pathways, and summarizes the mitochondrial dysfunction, lipid oxidation, insulin signaling pathway, inflammatory response, as well as its related pathways are instrumental in regulating T2DM.

The human gut microbiome is a complex ecosystem, composed of bacteria, archaea, fungi, viruses and protozoa in the human intestinal tract, which participate in material and energy metabolism, each exerting a unique influence on host metabolism (34, 35). In the context of T2DM, the gut microbiome exhibits notable changes, termed dysbiosis, which is closely related with dysregulation of a host metabolism, where there is an increase in bacteria that negatively impact metabolic health and a decrease in beneficial bacteria (36, 37). Bacteroides uniformis and Bacteroides acidifaciens can negatively improve glucose tolerance and insulin sensitivity, and are instrumental in managing T2DM, Faecalibacterium, Akkermansia, and Roseburia also exhibit similar negative correlations with the metabolic diseases (38, 39). Therefore, diabetic patients often exhibit an increase in harmful bacteria, such as Escherichia and Prevotella, Iatcu OC (40). Short-chain amino acids (SCFAs),like butyrate, propionate and acetate regulate pancreatic beta-cell activity, reduce hepatic gluconeogenesis, and modulate immune system functions (41). Moreover, the alternation of branched-chain amino acids (BCAAs) increases liver gluconeogenesis and inhibits liver adipogenesis, directly contributes to the pathogenesis of T2DM (42, 43). Notably, the changes in gut microbiota are related to LPS-induced inflammatory responses, exacerbating IR in T2DM,further emphasizing microbiome’s role in glucose homeostasis and immune response (44) (**Figure 2a**). This emerging field offers diverse applications, particularly in modifying the gut environment through the administration of probiotics, including Bifidobacterium (adolescentis, animalis, bifidum, reuteri, breve, longum)and Lactobacillus (acidophilus, casei, fermentum, gasseri, johnsonii, paracasei, plantarum, rhamnosus, and salivarius) can potentially alleviate or even reverse the metabolic dysfunctions associated with T2DM (45). Additionally, diabetic medications Alpha-glucosidase inhibitors promote the growth of beneficial microbes such as Bacteroides, Lactobacillus, and Faecalibacterium, while reducing populations of potentially pathogenic bacteria like Ruminococcus and Butyricicoccus (46). GLP-1 agonists also promote the growth of SCFA-producing bacteria such as Bifidobacterium and Bacteroides, further supporting glycemic control and metabolic health (47). Sodium-glucose co-transporter type 2 inhibitors, such as sotagliflozin, impact the gut microbiome by decreasing the Firmicutes/Bacteroides ratio and enhancing fatty acid production (48). Spermidine can significantly change the composition and function of intestinal microbiota, moderately reduce the level of circulating LPS, improve metabolic endotoxemia, weaken cell apoptosis to enhance intestinal barrier function (49). Fecal microbiota transplantation (FMT) effectively mitigates damage to the intestinal barrier through altering the microbial structure (50).

### 2.5 Inflammation

T2DM patients have elevated blood glucose and free fatty acids levels, dyslipidemia, impaired insulin receptor function. Metabolic inflammation is one of markedly causatives among the metabolic derangement factors (51). Specifically, chronic hyperglycemia and hyperlipidemia are the typical features of diabetes manifestations, which inevitably lead to glucolipotoxicity and in turn alter mitochondrial function. Dysfunctional mitochondria induce non-physiological generation of ROS, exacerbate the disturbance of inflammatory microenvironmental balance between adipose and pancreatic islet tissue, inevitably forming a vicious cycle (52, 53). Along with chronic production of proinflammatory cytokines and inefficient fatty acid β-oxidation, triggering a decrease in β cell insulin secretion and an increase in IR. Chronic overload of free fatty acids and glucose trigger inflammatory pathways (AMPK and PPARγ) directly or via increased production of ROS, which is the possible causative factor in the metabolic inflammation (54, 55),depicted in **Figure 2b**. Adipose tissue-derived cytokines, such as tumor necrosis factor-alpha (TNF-α) and interleukin-6 (IL-6), promote IR by interfering with insulin signaling pathway and promoting inflammation (56). The anti-inflammatory mediators, such as visfatin, plasminogen activator inhibitor-1, blocking IL-1R,anti-IL-1β,anti-TNF-α,CCR2 antagonists and IL-6R inhibitors, have emerged as available therapeutic approach for reversing T2DM (57).

### 2.6 Oxidative stress

Reactive oxygen species(ROS) are byproducts of mitochondrial metabolism, as hyperglycemia advances, oxidative stress in β-cells is often driven by mitochondrial ROS, which overwhelms the body’s antioxidant defenses, leading to impairment of insulin signaling pathway and metabolic dysregulation, thus plays a significant role in the pathophysiology of T2DM and its associated complications causing IR (58, 59). Most antioxidants, like SS-31 (elamipretide) and SkQ1,that reduces oxidative stress and maintains mitochondrial function offer a potential treatment strategy for T2DM and its complications (60). Additionally, ROS-mediated oxidative stress activates stress kinases and pro-inflammatory pathways, such as nuclear factor κB (NF-κB),which stimulates the release of proinflammatory cytokines and further exacerbating β-cell dysfunction and IR (1) (**Figure 2c**). Quercetin, curcumin, resveratrol, vitamin C and vitamin D have emerged as critical mediators in this antioxidant process, linking nuclear factor erythroid 2-related factor 2 (Nrf2) pathway and inflammation signaling to counteract β-cell dysfunction in diabetes (61). Glutathione (GSH), a water-soluble antioxidant, plays a critical role in maintaining cellular homeostasis and mitigating oxidative stress, study shows that metformin, teneligliptin, and pioglitazone not only impacts GSH redox pathway but also preserves β-cell function (62, 63). Therapeutic strategies focusing on reducing oxidative stress, enhancing antioxidant defenses, and focusing on Mediterranean diet, such as vegetables, function and preventing diabetes progression (64). It becomes increasingly evident that therapeutic strategies focusing on targeting oxidative stress pathways and enhancing antioxidant defenses as promising therapeutic strategies to preserve β-cell function and prevent diabetic progression.

## 3 Conclusion and perspective

Type 2 diabetes mellitus stands as the most prevalent metabolic disease globally, characterized by intricate pathophysiological mechanism, including IR, impaired glucose homeostasis and β-cell dysfunction, genetic and environmental variables are also implicated in the etiology of T2DM. Considering multifactorial contributors of diabetes, such as mitochondrial dysfunction, obesity and gut microbiota, mutually reinforcing each other, and creating a vicious cycle that exacerbates both insulin sensitivity and resistance, underscores the urgent need for effective T2DM management strategies that not only offer improved hypoglycemic control but also address the broader health implications (5). Retatrutide is a novel triple agonist of the glucose-dependent insulinotropic polypeptide, glucagon-like peptide 1 and glucagon receptors, distinguishes itself for its favorable glycemic control ability and its broader effects such as increasing insulin secretion, improving glucose homeostasis, and refining appetite modulation, clinical trials have demonstrated significant reductions in body weight superior to dulaglutide, tirzepatide, and even semaglutide (65–67). Dorzagliatin and Cadisegliatin are promising antidiabetic drugs activate Glucokinase (GK), which regulate glucose metabolism and enhance beta cell function in diabetic patients, although they are still in the clinical stages (68, 69).

As diabetes management continues to advance, regenerative medicine has evolved significantly, with modern therapeutic avenues and a broader focus on immunomodulatory properties and adjuvant delivery biomaterials to repair damaged tissues, like mesenchymal stem cells (70). Immune cell-derived exosomes (including engineered exosomes) may also regulate the function of immune cells by transferring miRNA and mRNA, making them a promising directions reverse metabolic disorders of T2DM (71). Although they have great clinical application prospects, there are associated hurdles about immune rejection, tumorigenesis and the precise manipulation of stem cell behaviors needed to be surmounted (70). Gene therapy differs from a glucocentric approach, utilizes lentivirus, adenovirus, adeno-associated virus (AAV), along with non-viral techniques like liposomes and naked DNA, to deliver the insulin gene to target tissues, such as CRISPR-Cas9 sequencing enables to precise modification mtDNA mutations associated with T2DM, offering potential therapeutic precision and safety for metabolic disorders (72, 73).

Omic-related fields have illuminated a path of immense promise and significant potential for revolutionizing modern therapeutic interventions. Insist hyperglycemia impacts the metabolism of glucose, lipids, amino acids, as well as gut microbiota, which all in turn impact the drug responses of individuals living with T2DM.Hence, metabolomics, lipidomics and microbiomics are applied to uncover additional biomarkers that might better predict heterogeneity observed in personalized management outcome and enhance our understanding of disease mechanisms, thus offering new opportunities for various diabetic stages and patient-specific therapeutic interventions (74). Additionally, with the complex nature of T2DM and its widespread prevalence, the integration of precision medicine into all-around care should consider a myriad of factors encompass social, demographics, phenotypic, biochemical, genetic aspects, and individual patient variability (75). Complementary and alternative medicine (CAM) therapies herbal remedies like cinnamon, fenugreek, and bitter melon, allied with mindbody therapies, including yoga, tai chi, and meditation offers holistic and patient-centered approach to alleviate disease progression, have been viewed as complementary strategy to advance T2DM care (2).Moreover, interdisciplinary research is the foreword trend to develop diabetic treatment, artificial intelligence (AI) technologies analyze genes, proteins and metabolites, provide more intelligent and precise support from early screening, diagnosis to personalized treatment and monitoring, and leveraging machine learning algorithms to assess the risk trend in metabolic diseases and empower patients in self-management (76, 77). While AI has opened new options for conquering complexity of the human insulin system, sole reliance on it for personalized treatment is far from enough, it still needs collaboration with healthcare professionals, researchers, and technical implementation experts to harness the full potential of AI in diabetic management (78).

## Data Availability

The original contributions presented in the study are included in the article/supplementary material. Further inquiries can be directed to the corresponding authors.

## References

[1] Caturano A D’Angelo M Mormone A Russo V Mollica MP Salvatore T. Oxidative stress in type 2 diabetes: impacts from pathogenesis to lifestyle modifications. Curr Issues Mol Biol. (2023) 45:6651–66. doi: 10.3390/cimb45080420 PMC10453126 37623239

[2] McBenedict B Orfao AL Goh KS Yau RCC Alphonse B Machado Lima J. The role of alternative medicine in managing type 2 diabetes: A comprehensive review. Cureus. (2024) 16:e61965. doi: 10.7759/cureus.61965 38978922 PMC11229830

[3] Cannon A Handelsman Y Heile M Shannon M. Burden of illness in type 2 diabetes mellitus. J Manag Care Spec Pharm. (2018) 24:S5–S13. doi: 10.18553/jmcp.2018.24.9-a.s5 30156443 PMC10408423

[4] Marx N Federici M Schütt K Müller-Wieland D Ajjan RA Antunes MJ. 2023 ESC Guidelines for the management of cardiovascular disease in patients with diabetes. Eur Heart J. (2023) 44:4043–140. doi: 10.1093/eurheartj/ehad192 37622663

[5] Caturano A Galiero R Rocco M Tagliaferri G Piacevole A Nilo D. Modern challenges in type 2 diabetes: balancing new medications with multifactorial care. Biomedicines. (2024) 12:2039. doi: 10.3390/biomedicines12092039 39335551 PMC11429233

[6] Vicente AM Ballensiefen W Jönsson JI. How personalised medicine will transform healthcare by 2030: the ICPerMed vision. J Transl Med. (2020) 18:180. doi: 10.1186/s12967-020-02316-w 32345312 PMC7189458

[7] Anderson S Bankier AT Barrell BG de Bruijn MH Coulson AR Drouin J. Sequence and organization of the human mitochondrial genome. Nature. (1981) 290:457–65. doi: 10.1038/290457a0 7219534

[8] Nolfi-Donegan D Braganza A Shiva S. Mitochondrial electron transport chain: Oxidative phosphorylation, oxidant production, and methods of measurement. Redox Biol. (2020) 37:101674. doi: 10.1016/j.redox.2020.101674 32811789 PMC7767752

[9] Luo JS Ning JQ Chen ZY Li WJ Zhou RL Yan RY. The role of mitochondrial quality control in cognitive dysfunction in diabetes. Neurochem Res. (2022) 47:2158–72. doi: 10.1007/s11064-022-03631-y PMC9352619 35661963

[10] Martinez J Marmisolle I Tarallo D Quijano C. Mitochondrial bioenergetics and dynamics in secretion processes. Front Endocrinol. (2020) 11:319. doi: 10.3389/fendo.2020.00319 PMC7256191 32528413

[11] Thubron EB Rosa HS Hodges A Sivaprasad S Francis PT Pienaar IS. Regional mitochondrial DNA and cell-type changes in post-mortem brains of non-diabetic Alzheimer’s disease are not present in diabetic Alzheimer’s disease. Sci Rep. (2019) 9:11386. doi: 10.1038/s41598-019-47783-4 31388037 PMC6684616

[12] Yang L Han W Luo Y Hu X Xu Y Li H. Adapentpronitrile, a new dipeptidyl peptidase-IV inhibitor, ameliorates diabetic neuronal injury through inhibiting mitochondria-related oxidative stress and apoptosis. Front Cell Neurosci. (2018) 12:214. doi: 10.3389/fncel 30072873 PMC6058014

[13] Ipsen EØ Madsen KS Chi Y Pedersen-Bjergaard U Richter B Metzendorf MI. Pioglitazone for prevention or delay of type 2 diabetes mellitus and its associated complications in people at risk for the development of type 2 diabetes mellitus. Cochrane Database Syst Rev. (2020) 11:CD013516. doi: 10.1002/14651858.CD013516.pub2 33210751 PMC8092670

[14] Hallakou-Bozec S Vial G Kergoat M Fouqueray P Bolze S Borel AL. Mechanism of action of Imeglimin: A novel therapeutic agent for type 2 diabetes. Diabetes Obes Metab. (2021) 23:664–73. doi: 10.1111/dom PMC8049051 33269554

[15] Detaille D Vial G Borel AL Cottet-Rouselle C Hallakou-Bozec S Bolze S. Imeglimin prevents human endothelial cell death by inhibiting mitochondrial permeability transition without inhibiting mitochondrial respiration. Cell Death Discov. (2016) 2:15072. doi: 10.1038/cddiscovery.2015.72 27551496 PMC4979505

[16] Sultana MA Hia RA Akinsiku O Hegde V. Peripheral mitochondrial dysfunction: A potential contributor to the development of metabolic disorders and alzheimer’s disease. Biol (Basel). (2023) 12:1019. doi: 10.3390/biology12071019 PMC10376519 37508448

[17] Rius-Pérez S Torres-Cuevas I Millán I Ortega ÁL Pérez S. PGC-1*α*, inflammation, and oxidative stress: an integrative view in metabolism. Oxid Med Cell Longev. (2020) 2020:1452696. doi: 10.1155/2020/1452696 32215168 PMC7085407

[18] Tang BL. Sirt1 and the mitochondria. Mol Cells. (2016) 39:87–95. doi: 10.14348/molcells.2016.2318 26831453 PMC4757807

[19] Galicia-Garcia U Benito-Vicente A Jebari S Larrea-Sebal A Siddiqi H Uribe KB. Pathophysiology of type 2 diabetes mellitus. Int J Mol Sci. (2020) 21:6275. doi: 10.3390/ijms21176275 32872570 PMC7503727

[20] Golubitzky A Dan P Weissman S Link G Wikstrom JD Saada A. Screening for active small molecules in mitochondrial complex I deficient patient’s fibroblasts, reveals AICAR as the most beneficial compound. PloS One. (2011) 6:e26883. doi: 10.1371/journal.pone.0026883 22046392 PMC3202581

[21] Russell OM Gorman GS Lightowlers RN Turnbull DM. Mitochondrial diseases: hope for the future. Cell. (2020) 181:168–88. doi: 10.1016/j.cell.2020.02.051 32220313

[22] Szűcs G Pipicz M Szabó MR Csont T Török L Csonka C. Effect of eccentric exercise on metabolic health in diabetes and obesity. Sports Med Open. (2023) 9:91. doi: 10.1186/s40798-023-00596-2 37775653 PMC10541389

[23] Nussbaumerova B Rosolova H. Obesity and dyslipidemia. Curr Atheroscler Rep. (2023) 25:947–55. doi: 10.1007/s11883-023-01167-2 37979064

[24] Xu JQ Jiang MX Xu YJ Dong SJ. Research progress in the regulation of functional homeostasis of adipose tissue by exosomal miRNA. Sheng Li Xue Bao. (2024) 76:791–800. 39468815

[25] Wankhade UD Shen M Yadav H Thakali KM. Novel browning agents, mechanisms, and therapeutic potentials of brown adipose tissue. BioMed Res Int. (2016) 2016:2365609. doi: 10.1155/2016/2365609 28105413 PMC5220392

[26] Ghesmati Z Rashid M Fayezi S Gieseler F Alizadeh E Darabi M. An update on the secretory functions of brown, white, and beige adipose tissue: Towards therapeutic applications. Rev Endocr Metab Disord. (2024) 25:279–308. doi: 10.1007/s11154-023-09850-0 38051471 PMC10942928

[27] de Marañón AM Díaz-Pozo P Canet F Díaz-Morales N Abad-Jiménez Z López-Domènech S. Metformin modulates mitochondrial function and mitophagy in peripheral blood mononuclear cells from type 2 diabetic patients. Redox Biol. (2022) 53:102342. doi: 10.1016/j.redox.2022.102342 35605453 PMC9124713

[28] Fatima A Rasool S Devi S Talha M Waqar F Nasir M. Exploring the cardiovascular benefits of sodium-glucose cotransporter-2 (SGLT2) inhibitors: expanding horizons beyond diabetes management. Cureus. (2023) 15:e46243. doi: 10.7759/cureus.46243 37908957 PMC10613932

[29] Tomita I Kume S Sugahara S Osawa N Yamahara K Yasuda-Yamahara M. SGLT2 inhibition mediates protection from diabetic kidney disease by promoting ketone body-induced mTORC1 inhibition. Cell Metab. (2020) 32:404–419.e6. doi: 10.1016/j.cmet.2020.06.020 32726607

[30] Cai X Yang W Gao X. The association between the dosage of SGLT2 inhibitor and weight reduction in type 2 diabetes patients: A meta-analysis. Obesity. (2018) 26:70–80:et al. doi: 10.1002/oby.22066 29165885

[31] Thomas MK Nikooienejad A Bray R Cui X Wilson J Duffin K. Dual GIP and GLP-1 receptor agonist tirzepatide improves beta-cell function and insulin sensitivity in type 2 diabetes. J Clin Endocrinol Metab. (2021) 106:388–96. doi: 10.1210/clinem/dgaa863 PMC7823251 33236115

[32] Zimmermann T Thomas L Baader-Pagler T Haebel P Simon E Reindl W. BI 456906: Discovery and preclinical pharmacology of a novel GCGR/GLP-1R dual agonist with robust anti-obesity efficacy. Mol Metab. (2022) 66:101633. doi: 10.1016/j.molmet.2022.101633 36356832 PMC9679702

[33] Kermansaravi M Chiappetta S Kassir R Bosco A Giudicelli X Lainas P. Efficacy of one anastomosis gastric bypass versus sleeve gastrectomy and roux-en-Y gastric bypass for the treatment of type 2 diabetes mellitus: a systematic review and meta-analysis of randomized clinical trials. Obes Surg. (2024) 34:4555–62. doi: 10.1007/s11695-024-07564-z 39496986

[34] Wang R Tang R Li B Ma X Schnabl B Tilg H. Gut microbiome, liver immunology, and liver diseases. Cell Mol Immunol. (2021) 18:4–17. doi: 10.1038/s41423-020-00592-6 33318628 PMC7852541

[35] Zhou Z Sun B Yu D Zhu C. Gut microbiota: an important player in type 2 diabetes mellitus. Front Cell Infect Microbiol. (2022) 12:834485. doi: 10.3389/fcimb.2022.834485 35242721 PMC8886906

[36] Mercer K Yeruva L Pack L Graham JL Stanhope KL Chintapalli SV. Xenometabolite signatures in the UC davis type 2 diabetes mellitus rat model revealed using a metabolomics platform enriched with microbe-derived metabolites. American journal of physiology. Gastrointest. Liver. Physiol. (2020) 319:G157–69. doi: 10.1152/ajpgi.00105.2020 PMC7500265 32508155

[37] Noureldein M Bitar S Youssef N Azar S Eid AA. Butyrate modulates diabetes-linked gut dysbiosis: epigenetic and mechanistic modifications. J Mol Endocrinol. (2020) 64:29–42. doi: 10.1530/jme-19-0132 31770101

[38] Yang JY Lee YS Kim Y Lee SH Ryu S Fukuda S. Gut commensal Bacteroides acidifaciens prevents obesity and improves insulin sensitivity in mice. Mucosal Immunol. (2017) 10:104–16. doi: 10.1038/mi.2016.42 27118489

[39] Zhang X Shen D Fang Z Jie Z Qiu X Zhang C. Human gut microbiota changes reveal the progression of glucose intolerance. PloS One. (2013) 8:e71108. doi: 10.1371/journal.pone.0071108 24013136 PMC3754967

[40] Hamamah S Covasa M. Harnessing prebiotics to improve type 2 diabetes outcomes. Nutrients. (2024) 16:3447. doi: 10.3390/nu16203447 39458444 PMC11510484

[41] Jeyaraman M Mariappan T Jeyaraman N Muthu S Ramasubramanian S Santos GS. Gut microbiome: A revolution in type II diabetes mellitus. World J Diabetes. (2024) 15:1874–88. doi: 10.4239/wjd.v15.i9.1874 PMC11372632 39280189

[42] Vanweert F de Ligt M Hoeks J Hesselink MKC Schrauwen P Phielix E. Elevated plasma branched-chain amino acid levels correlate with type 2 diabetes-related metabolic disturbances. J Clin Endocrinol Metab. (2021) 106:e1827–1836:et al. doi: 10.1210/clinem/dgaa751 33079174

[43] Zhao H Zhang F Sun D Wang X Zhang X Zhang J. Branched-chain amino acids exacerbate obesity-related hepatic glucose and lipid metabolic disorders via attenuating akt2 signaling. Diabetes. (2020) 69:1164–77. doi: 10.2337/db19-0920 32184272

[44] Fang Y Zhang C Shi H Wei W Shang J Zheng R. Characteristics of the gut microbiota and metabolism in patients with latent autoimmune diabetes in adults: A case-control study. Diabetes Care. (2021) 44:2738–46. doi: 10.2337/dc20-2975 PMC8669532 34620611

[45] Rittiphairoj T Pongpirul K Janchot K Mueller NT Li T. Probiotics contribute to glycemic control in patients with type 2 diabetes mellitus: a systematic review and meta-analysis. Adv Nutr. (2021) 12:722–34. doi: 10.1093/advances/nmaa133 PMC8166562 33126241

[46] Zhang X Fang Z Zhang C Xia H Jie Z Han X. Effects of acarbose on the gut microbiota of prediabetic patients: A randomized, double-blind, controlled crossover trial. Diabetes Ther. (2017) 8:293–307. doi: 10.1007/s13300-017-0226-y 28130771 PMC5380489

[47] Zhang Q Xiao X Zheng J Li M Yu M Ping F. Featured article: Structure moderation of gut microbiota in liraglutide-treated diabetic male rats. Exp Biol Med. (2018) 243:34–44. doi: 10.1177/1535370217743765 PMC5788162 29171288

[48] Cefalo CMA Cinti F Moffa S Impronta F Sorice GP Mezza T. Sotagliflozin, the first dual SGLT inhibitor: current outlook and perspectives. Cardiovasc Diabetol. (2019) 18:20. doi: 10.1186/s12933-019-0828-y 30819210 PMC6393994

[49] Eisenberg T Knauer H Schauer A Büttner S Ruckenstuhl C Carmona-Gutierrez D. Induction of autophagy by spermidine promotes longevity. Nat Cell Biol. (2009) 11:1305–14. doi: 10.1038/ncb1975 19801973

[50] Cheng S Ma X Geng S Jiang X Li Y Hu L. Fecal microbiota transplantation beneficially regulates intestinal mucosal autophagy and alleviates gut barrier injury. mSystems. (2018) 3:e00137–18. doi: 10.1128/mSystems.00137-18 PMC6178585 30320222

[51] Arabshomali A Bazzazzadehgan S Mahdi F Shariat-Madar Z. Potential benefits of antioxidant phytochemicals in type 2 diabetes. Molecules. (2023) 28:7209. doi: 10.3390/molecules28207209 37894687 PMC10609456

[52] Tangvarasittichai S. Oxidative stress, insulin resistance, dyslipidemia and type 2 diabetes mellitus. World J Diabetes. (2015) 6:456–80. doi: 10.4239/wjd.v6.i3.456 PMC4398902 25897356

[53] Gao Z Chen X. Fatty acid β-oxidation in kidney diseases: perspectives on pathophysiological mechanisms and therapeutic opportunities. Front.Pharmacol. (2022) 13:805281. doi: 10.3389/fphar.2022.805281 35517820 PMC9065343

[54] Pawlak M Lefebvre P Staels B. Molecular mechanism of PPARα action and its impact on lipid metabolism, inflammation and fibrosis in non-alcoholic fatty liver disease. J Hepatol. (2015) 62:720–33. doi: 10.1016/j.jhep.2014.10.039 25450203

[55] Ke R Xu Q Li C Luo L Huang D. Mechanisms of AMPK in the maintenance of AT balance during energy metabolism. Cell Biol Int. (2018) 42:384–92. doi: 10.1002/cbin.10915 29205673

[56] Iheagwam FN Batiha GE Ogunlana OO Chinedu SN. *Terminalia catappa* extract palliates redox imbalance and inflammation in diabetic rats by upregulating nrf-2 gene. Int J Inflam. (2021) 2021:9778486. doi: 10.1155/2021/9778486 34956587 PMC8702315

[57] Dakroub A Nasser S A Younis N Bhagani H Al-Dhaheri Y Pintus G. Visfatin: A possible role in cardiovasculo-metabolic disorders cells. Cells. (2020) 9:2444. doi: 10.3390/cells9112444 33182523 PMC7696687

[58] S S Hegde SV Agarwal SV Ns D Pillai A Shah SN. Biomarkers of oxidative stress and their clinical relevance in type 2 diabetes mellitus patients: A systematic review. Cureus. (2024) 16:e66570. doi: 10.7759/cureus.66570 39252730 PMC11382618

[59] Iheagwam FN Iheagwam OT Onuoha MK Ogunlana OO Chinedu SN. Terminalia catappa aqueous leaf extract reverses insulin resistance, improves glucose transport and activates PI3K/AKT signalling in high fat/streptozotocin-induced diabetic rats. Sci Rep. (2022) 12:10711. doi: 10.1038/s41598-022-15114-9 35739183 PMC9226017

[60] Mason SA Wadley GD Keske MA Parker L. Effect of mitochondrial-targeted antioxidants on glycaemic control, cardiovascular health, and oxidative stress in humans: A systematic review and meta-analysis of randomized controlled trials. Diabetes Obes Metab. (2022) 24:1047–60. doi: 10.1111/dom.14669 PMC9314850 35165982

[61] Veluthakal R Esparza D Hoolachan JM Balakrishnan R Ahn M Oh E. Mitochondrial dysfunction, oxidative stress, and inter-organ miscommunications in T2D progression. Int J Mol Sci. (2024) 25:1504. doi: 10.3390/ijms25031504 38338783 PMC10855860

[62] Karunakaran U Elumalai S Moon JS Won KC. Pioglitazoneinduced AMPK-glutaminase-1 prevents high glucose-induced pancreatic β-cell dysfunction by glutathione antioxidant system. Redox Biol. (2021) 45:102029. doi: 10.1016/j.redox.2021.102029 34107382 PMC8187239

[63] Moon JS Karunakaran U Elumalai S Lee IK Lee HW Kim YW. Metformin prevents glucotoxicity by alleviating oxidative and ER stress-induced CD36 expression in pancreatic beta cells. J Diabetes Complications. (2017) 31:21–30. doi: 10.1016/j.jdiacomp.2016.09.001 27662780

[64] Caturano A Rocco M Tagliaferri G Piacevole A Nilo D Di Lorenzo G. Oxidative stress and cardiovascular complications in type 2 diabetes: from pathophysiology to lifestyle modifications. Antioxidants. (2025) 14:72. doi: 10.3390/antiox14010072 39857406 PMC11759781

[65] Abdul-Rahman T Roy P Ahmed FK Mueller-Gomez JL Sarkar S Garg N. The power of three: Retatrutide’s role in modern obesity and diabetes therapy. Eur J Pharmacol. (2024) 985:177095. doi: 10.1016/j.ejphar.2024.177095 39515565

[66] Sanyal AJ Kaplan LM Frias JP Brouwers B Wu Q Thomas MK. Triple hormone receptor agonist retatrutide for metabolic dysfunction-associated steatotic liver disease: a randomized phase 2a trial. Nat Med. (2024) 30:2037–48. doi: 10.1038/s41591-024-03018-2 PMC11271400 38858523

[67] Caturano A Galiero R Loffredo G Vetrano E Medicamento G Acierno C. Effects of a combination of empagliflozin plus metformin vs. Metformin monotherapy on NAFLD progression in type 2 diabetes: the IMAGIN pilot study. Biomedicines. (2023) 11:322. doi: 10.3390/biomedicines11020322 36830859 PMC9952909

[68] Wang P Liu H Chen L Duan Y Chen Q Xi S. Effects of a novel glucokinase activator, HMS5552, on glucose metabolism in a rat model of type 2 diabetes mellitus. J Diabetes Res. (2017) 2017:5812607. doi: 10.1155/2017/5812607 28191470 PMC5278194

[69] Vella A Freeman JLR Dunn I Keller K Buse JB Valcarce C. Targeting hepatic glucokinase to treat diabetes with TTP399, a hepatoselective glucokinase activator. Sci Transl Med. (2019) 11:eaau3441. doi: 10.1126/scitranslmed.aau3441 30651321

[70] Raoufinia R Rahimi HR Saburi E Moghbeli M. Advances and challenges of the cell-based therapies among diabetic patients. J Transl Med. (2024) 22:435. doi: 10.1186/s12967-024-05226-3 38720379 PMC11077715

[71] Ma X Yao M Gao Y Yue Y Li Y Zhang T. Functional immune cellderived exosomes engineered for the trilogy of radiotherapy sensitization. Adv Sci. (2022) 9:e2106031. doi: 10.1002/advs.202106031 PMC9376809 35715382

[72] Kar B Castillo SR Sabharwal A Clark KJ Ekker SC. Mitochondrial base editing: recent advances towards therapeutic opportunities. Int J Mol Sci. (2023) 24:5798. doi: 10.3390/ijms24065798 36982871 PMC10056815

[73] Wong MS Hawthorne WJ Manolios N. Gene therapy in diabetes. Self Nonself. (2010) 1:165. doi: 10.4161/self.1.3.12643 21487475 PMC3047781

[74] Rakusanova S Cajka T. Metabolomics and lipidomics for studying metabolic syndrome: insights into cardiovascular diseases, type 1 & 2 diabetes, and metabolic dysfunction-associated steatotic liver disease. Physiol Res. (2024) 73:S165–83. doi: 10.33549/physiolres.935443 PMC11412346 39212142

[75] Oh SH Lee SJ Park J. Precision medicine for hypertension patients with type 2 diabetes via reinforcement learning. J Pers Med. (2022) 12:87. doi: 10.3390/jpm12010087 35055402 PMC8781402

[76] Hsu NW Chou KC Wang YT Hung CL Kuo CF Tsai SY. Building a model for predicting metabolic syndrome using artificial intelligence based on an investigation of whole-genome sequencing. J Transl Med. (2022) 20:190. doi: 10.1186/s12967-022-03379-7 35484552 PMC9052619

[77] Maiese K. Diabetes mellitus and glymphatic dysfunction: Roles for oxidative stress, mitochondria, circadian rhythm, artificial intelligence, and imaging. World J Diabetes. (2025) 16:98948. doi: 10.4239/wjd.v16.i1.98948 39817214 PMC11718455

[78] Tahir F Farhan M. Exploring the progress of artificial intelligence in managing type 2 diabetes mellitus: a comprehensive review of present innovations and anticipated challenges ahead. Front Clin Diabetes Healthc. (2023) 4:1316111. doi: 10.3389/fcdhc.2023.1316111 38161783 PMC10757318

